# Adsorptive desulfurization of DBT/*n*-decane using UiO-66-functionalized silica and γ-alumina porous ceramic membranes

**DOI:** 10.1039/d6ra05929e

**Published:** 2026-09-07

**Authors:** El-Sayed M. El-Sayed, Hussien A. El Sayed, Ahmed M. A. El Naggar, M. A. Sayed, Mahmoud F. Mubarak, Ahmed Shahat, Reda M. Abd El-Aal

**Affiliations:** a Refining Department, Egyptian Petroleum Research Institute Nasr City Cairo 11727 Egypt elsayedm.elsayed@hotmail.com; b Petroleum Applications Department, Egyptian Petroleum Research Institute (EPRI) Ahmed El-Zomor, Nasr City Cairo 11727 Egypt; c Core Lab Center, Egyptian Petroleum Research Institute (EPRI) 1 Ahmed El Zomor St., Nasr City Cairo 11727 Egypt; d Department of Chemistry, Faculty of Science, Suez University Suez 3518 Egypt; e Chemistry Department, Faculty of Science, Islamic University of Madinah Madinah 42351 Saudi Arabia

## Abstract

Metal–organic frameworks (MOFs) are promising sulfur adsorbents, but fine powders are hard to recover and handle continuously. Here, UiO-66 was incorporated into silica- and γ-alumina-based PVA-bound ceramic-composite membranes at nominal loadings of 1, 3, and 5 wt% and evaluated for dibenzothiophene (DBT) removal from *n*-decane. The 3 wt% formulations maximized both adsorption capacity and sulfur rejection within the tested range and were therefore selected for the subsequent operating-parameter studies. Detailed characterization of the silica blank (S-0), alumina blank (A-0), UiO-66@silica (S-3), and UiO-66@alumina (A-3) by XRD, FTIR, SEM, N_2_ physisorption, TGA, and ICP-OES confirmed 2.98/2.91 wt% UiO-66 loading. BET surface area increased from 300 → 324 m^2^ g^−1^ (silica) and 199 → 226 m^2^ g^−1^ (alumina), with type IV mesoporosity retained alongside added UiO-66 microporosity. In recirculating cross-flow tests at 25 °C, S-3 performed best, reaching 25.0 mg S per g uptake, 62.5% DBT rejection, and 157.5 L m^−2^ h^−1^ flux at 120 min while retaining 88% capacity after five methanol–regeneration cycles. UiO-66-functionalized ceramic-composite membranes are thus promising, reusable materials for adsorptive desulfurization.

## Abstract 2

Robust UiO-66-functionalized ceramic-composite membranes achieve effective continuous adsorptive desulfurization of DBT/*n*-decane with high sulfur rejection and cycling stability.

## Graphical abstract

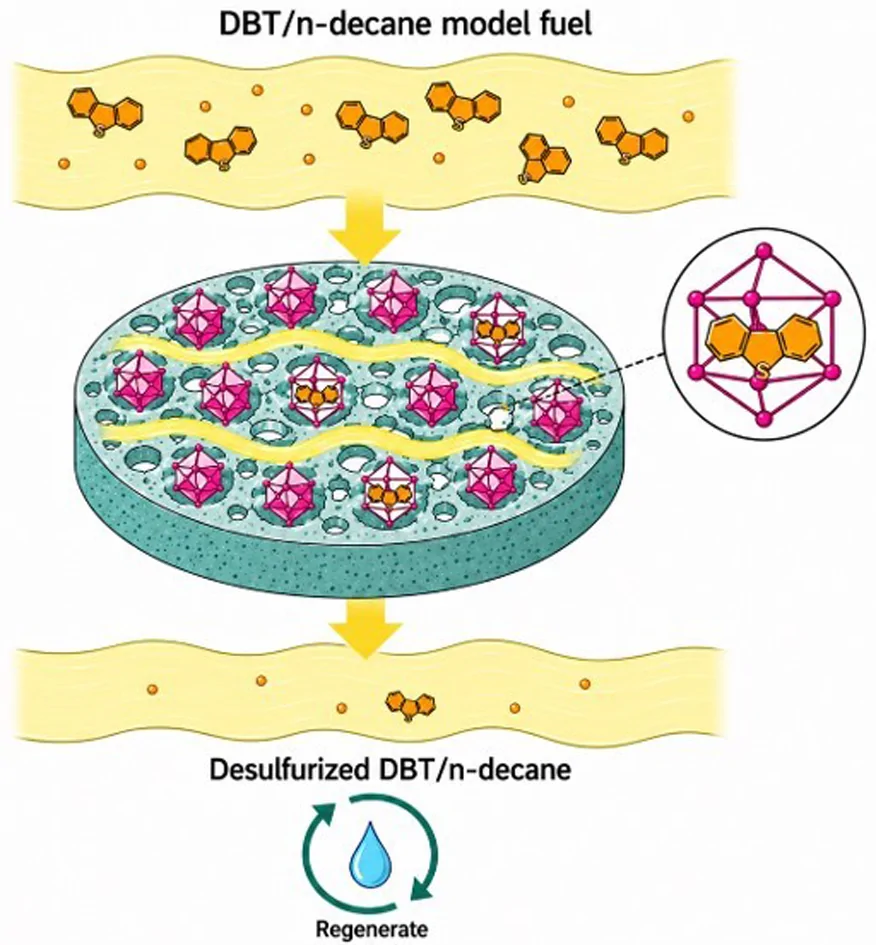

## 1. Introduction

Sulfur compounds are major fuel contaminants: combustion forms SOx (acid deposition, particulates) and they poison noble-metal catalysts downstream. Regulation has progressively cut fuel sulfur limits to today's ultra-low standard, <10–15 ppm.^1,2^ Delivering these targets cost-effectively is now central to refining and fuel-treatment operations.

Hydrodesulfurization (HDS) remains the industrial workhorse for sulfur abatement, but becomes less effective as refractory species dominate the residual sulfur. Steric hindrance around the sulfur atom on conventional Co–Mo/Ni–Mo catalysts forces higher operating temperature, pressure and reactor volume, raising hydrogen consumption and capital cost.^3,4^

Adsorptive desulfurization (ADS) is an increasingly popular non-hydrogenative option, operating near ambient conditions without hydrogen and integrable as a downstream refinery process.^5,6^ Performance depends on adsorbent structure/chemistry, shifting interest toward tailorable porous crystalline materials for selective, high-capacity dibenzothiophene (DBT) uptake. Recent comprehensive surveys of metal–organic frameworks (MOF)-based fuel desulfurization confirm this shift and identify adsorptive routes as the most energy-efficient complement to conventional hydrotreatment,^6,7^ while a growing body of 2025–2026 studies – from machine-learning-guided screening of MOF adsorbents^8^ to new framework chemistries^9^ and engineered UiO-66 derivatives^10^ – continues to expand the material portfolio for refractory sulfur capture; recent reviews distil these into rational metal-node/linker design principles.^11,12^

MOFs suit this use: their pore chemistry is tunable *via* metal-node/linker choice, and their crystallinity enables predictable, site-specific adsorbate interactions.^13,14^ UiO-66 is a well-studied MOF for liquid-phase separations, with stable Zr_6_O_4_(OH)_4_ nodes, rigid BDC linkers, and microporous ∼8/∼11 Å cages accessed by ∼6 Å windows.^15,16^ Its Zr^4+^ centers and linkers interact with thiophenic sulfur *via* Lewis acid–base coordination, π–π stacking, and confinement. The high DBT capacity reported for UiO-66 and its analogues^17,18^ likely reflects DBT's size (∼9 × 7 × 4 Å) fitting UiO-66's cages closely enough to maximize dispersion forces. UiO-66 was chosen as a benchmark MOF whose reproducible synthesis and established DBT-binding chemistry let performance changes be attributed to shaping/deployment, not chemistry uncertainty.

However, using UiO-66 as a free powder in continuous operations is hindered by practical issues with fine MOF particles: solid/liquid separation, entrainment, channeling/pressure drop in packed beds, cycling agglomeration, and scale-up.^19^ Embedding the MOF in a structured support addresses this; membranes are of interest because the adsorbent and separation share one body, giving continuous adsorption–permeation without a separate filtration step.

Silica and γ-alumina ceramic membranes suit hosting UiO-66: they combine mechanical robustness with thermal and chemical resistance, are compatible with hydrocarbon feeds, and possess intrinsically mesoporous architectures that favor convective transport across the membrane body without imposing prohibitive flow resistance.^20,21^ This creates a hierarchical structure: host mesopores act as convective conduits while UiO-66 microporosity supplies high-affinity DBT sites, translating the MOF's capacity into a deployable format.

A silica- and alumina-based loading series comprising blanks and 1, 3, and 5 wt% UiO-66 formulations was first evaluated under the baseline operating conditions. The 3 wt% formulations were selected from the combined adsorption-capacity and sulfur-rejection responses. The four membranes carried forward—S-0, A-0, S-3, and A-3—were characterized by XRD, FTIR, SEM, N_2_ physisorption, and TGA and then evaluated at 25 °C for the effects of pressure (10–30 bar), concentration (50–200 mg L^−1^), and flow rate (0.25–1.0 L min^−1^) on capacity, flux, and rejection, together with cycling stability over five methanol regenerations. The objective was to establish structure–property–performance relationships and identify the more effective ceramic support and UiO-66 loading.

The literature on UiO-66-based desulfurization is dominated by two configurations: (a) batch adsorption on pristine/functionalized UiO-66 powders—optimized capacity/selectivity but a difficult-to-deploy fine solid^18,22,23^ and (b) oxidative desulfurization catalysis over UiO-66 derivatives, which requires an external oxidant and downstream sulfone removal.^10^ The most recent reviews of the field^6,7,11,12^ indicate that the bottleneck is no longer framework chemistry but shaping/integration into continuous, robust separation formats. Few reports couple adsorption with filtration in a single body, motivating the dual-host membrane approach pursued here.

Although UiO-66-based metal–organic frameworks have been extensively investigated as powder adsorbents for adsorptive desulfurization of model diesel fuels, their incorporation into structured, mechanically handleable membrane formats remains comparatively under-explored.^6,7,24^ Furthermore, the integration of UiO-66 into ceramic membrane supports – specifically silica and alumina – for fuel desulfurization has not been previously reported.^11,12^ This work introduces UiO-66/silica and UiO-66/alumina membranes for DBT/*n*-decane desulfurization, combining UiO-66's surface area/porosity with ceramic robustness. To our knowledge, this silica-*vs.*-alumina configuration is unreported, offering a route to membrane-based middle-distillate desulfurization; extension to real fuel streams is left for future work.

## 2. Experimental section

### 2.1. Materials and chemicals

ZrCl_4_ (≥99.5%), H_2_BDC (98%), DMF (≥99.8%), and HCl (37%)—sourced from Sigma-Aldrich—were used as received for UiO-66 synthesis. PVA (Mw ≈ 89 000–98 000, ≥99% hydrolyzed) was used as the casting binder. Mesoporous SiO_2_ (10–20 µm, BET ≈ 350 m^2^ g^−1^) and γ-Al_2_O_3_ (5–15 µm, BET ≈ 200 m^2^ g^−1^) were used as the ceramic matrices. DBT (98%) was used as the model sulfur compound; *n*-decane (≥99.9%) was used as the model-fuel/rinse solvent; methanol (≥99.9%) was used as the regeneration solvent. Deionized water was used throughout.

### 2.2. Synthesis of UiO-66

UiO-66 was synthesized *via* a modulated solvothermal route adapted from Cavka *et al.*^15^ For a typical batch, 1.25 g ZrCl_4_ and 0.89 g H_2_BDC (5.36 mmol each) were dissolved in 50 mL DMF and mixed, and 1.5 mL HCl was added dropwise as a modulator. The mixture was sonicated (20 min), autoclaved (150 mL Teflon-lined, 24 h, 120 °C), then centrifuged (8000 rpm, 10 min), washed 3× with DMF and 3× with methanol (30 mL each), and activated under vacuum (150 °C, 12 h) to give UiO-66 as an off-white powder.

### 2.3. Preparation of the ceramic membranes

Eight flat-sheet PVA-bound membrane formulations were doctor-blade cast: silica blank (S-0); silica composites containing nominal UiO-66 loadings of 1, 3, and 5 wt% (S-1, S-3, and S-5); alumina blank (A-0); and the corresponding alumina composites (A-1, A-3, and A-5). The numeral denotes the nominal UiO-66 loading as wt% of total solids. The 1 and 5 wt% formulations were prepared for loading screening, whereas detailed physicochemical characterization was performed on the blanks and the 3 wt% formulations selected for the subsequent operating-parameter studies. PVA was used as the retained binder and UiO-66 as the adsorptive phase; accordingly, the coupons are PVA-bound ceramic-composite films rather than fully sintered ceramic membranes. ICP-OES-confirmed UiO-66 contents of 2.98 and 2.91 wt% for S-3 and A-3, respectively, agreed with the nominal 3 wt% loading.

The 1 wt% PVA solution was prepared by dissolving PVA in water (85 °C, 4 h), followed by cooling and degassing. Silica or γ-alumina was dispersed for the blanks by sonication (40 kHz, 30 min) and stirring (500 rpm, 12 h). For the composite formulations, the required quantity of UiO-66 (1, 3, or 5 wt% of total solids) was pre-dispersed and sonicated for 30 min before addition to the ceramic/PVA slurry; the complete slurry was then sonicated for 1 h and stirred for 12 h to promote homogeneous dispersion.

For each batch, the total solid mass was 10.00 g. The blanks contained 9.70 g ceramic and 0.30 g PVA. For the 1, 3, and 5 wt% UiO-66 formulations, the corresponding ceramic/PVA/UiO-66 masses were 9.603/0.297/0.100 g, 9.409/0.291/0.300 g, and 9.215/0.285/0.500 g, respectively, thereby maintaining a 97 : 3 ceramic-to-PVA mass ratio within the non-MOF fraction. Each formulation was dispersed in approximately 29 mL water (approximately 26 wt% slurry solids). Three to five coupons were obtained from each cast sheet, with dry-mass variation ≤2%.

Slurries were cast using a doctor blade with a 300 µm gate (wet film), dried under ambient conditions (25 ± 2 °C, approximately 50% RH, 24 h), and then vacuum-dried stepwise at 60 °C for 6 h and 120 °C for 4 h. These temperatures were kept below 150 °C to preserve UiO-66. For the four membranes carried forward for detailed characterization, the cured 25 mm coupons (approximately 0.25 g) had thicknesses of 172, 168, 176, and 173 µm; porosities of 36.2, 33.5, 34.8, and 32.4%; and densities of 1.46, 1.62, 1.49, and 1.65 g cm^−3^ for S-0, A-0, S-3, and A-3, respectively. All coupons were immersed in *n*-decane for 30 min before each run to minimize gross air entrapment using a consistent preconditioning procedure.

### 2.4. Characterization

Detailed physicochemical characterization was performed on S-0, A-0, S-3, and A-3 after the loading-screening step. XRD (Bruker D8 Advance, Cu Kα, *λ* = 1.5406 Å; 2*θ* = 4–65°) probed structure and phase integrity. FTIR (4000–400 cm^−1^, KBr) identified the functional groups. SEM (JEOL JSM-7600F) examined the morphology of the prepared materials. N_2_ physisorption (77 K, ASAP 2020) provided BET surface area and pore-volume data. TGA (Q500, N_2_, 25–800 °C) assessed the thermal stability.

### 2.5. Model fuel (DBT in *n*-decane)

The model fuel is *n*-decane (hydrocarbon) plus DBT (sulfur compound), a single-component system for reproducible evaluation; multicomponent fuels are left for future work. Stock solution: 200 mg S per L (working-range upper limit, representative of post-HDS refractory sulfur); concentrations are total-sulfur basis. DBT was used as it is among the most difficult aromatic sulfur compounds to remove from middle-distillates, a stringent test.^3,4^ The stock was stirred (40 °C, 2 h) and stored in sealed amber bottles, dark, room temperature. Working solutions (50/100/150 mg L^−1^) were freshly diluted; 200 mg L^−1^ was used directly. Concentration was verified by UV-vis (≈325 nm) against the calibration curve.

### 2.6. Adsorption experiments

**Scheme 1:**
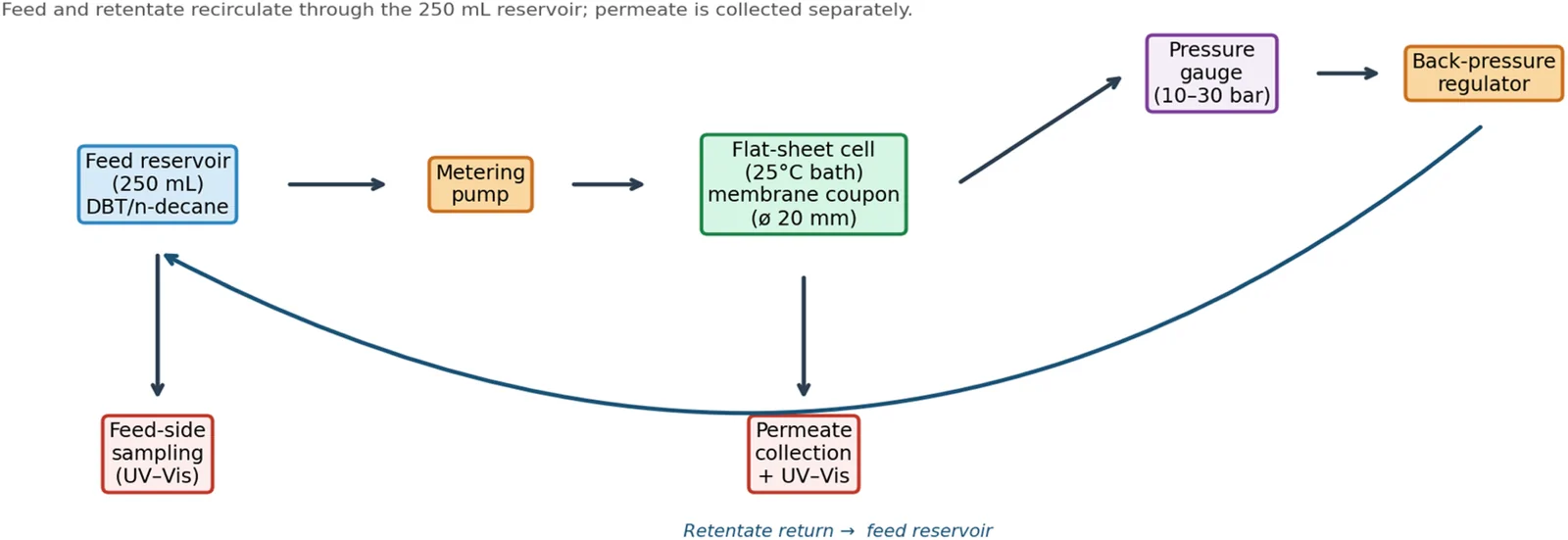
Recirculating cross-flow permeation set-up: DBT/*n*-decane fuel (250 mL reservoir) is pumped across the membrane coupon (20 mm) in a thermostatic cell (25 °C, 10–30 bar, 0.25–1.0 L min^−1^); retentate returns *via* a back-pressure regulator, and permeate/feed-side streams are sampled every 20 min and analyzed by UV-vis (∼325 nm).

Capacity and sulfur rejection were first measured for the silica and alumina loading series (blank, 1, 3, and 5 wt% UiO-66) under the baseline conditions of 25 °C, 20 bar, 0.5 L min^−1^, and 100 mg S per L, with sampling every 20 min for 120 min. The 3 wt% formulations were selected from this screening step based on the simultaneous capacity and rejection responses. Subsequent pressure, concentration, and flow-rate experiments were therefore performed using S-0, A-0, S-3, and A-3. In all tests, a 25 mm coupon (approximately 0.25 g) was placed between the feed and permeate compartments of a flat-sheet cross-flow cell with a 20 mm O-ring and supplied from a 250 mL reservoir by a calibrated pump; the retentate was returned through a back-pressure regulator (Scheme 1). Pressure (10–30 bar), flow rate (0.25–1.0 L min^−1^), and concentration (50–200 mg S per L) were varied independently over 120 min around the baseline condition. DBT was measured by UV-vis at approximately 325 nm (Section 2.5); the reported values therefore correspond to DBT-derived sulfur in the model fuel. The method was validated over 10–200 mg S per L (*R*^2^ = 0.9996; LOD = 0.85 mg S per L; LOQ = 2.58 mg S per L; RSD = 1.21%; recovery = 98.7%); a sulfur-specific technique such as GC-SCD is required for real multicomponent fuels.

The instantaneous adsorption capacity at time *t*, *q*_*t*_ (mg g^−1^), was calculated by a mass balance on the membrane (eqn (1)):

1 *q*_*t*_ = (*C*_0_ − *C*_*t*_) × *V*/*m*

where *C*_0_ (mg L^−1^) is the initial feed sulfur concentration, *C*_*t*_ (mg L^−1^) the concentration at time *t*, *V* (L) is the feed volume in contact with the membrane, and *m* (g) is the coupon mass (≈0.25 g). Capacities are normalized to total composite mass, reflecting the device-level metric relevant to membrane operation.

The fractional sulfur rejection, *R* (%), was determined from the simultaneous feed and permeate sulfur concentrations as follows:

2 *R* (%) = (*C*_n_ − *C*_p_)/*C*_n_ × 100

with *C*_n_ and *C*_p_ (mg L^−1^) the instantaneous feed- and permeate-side sulfur concentrations at the same time *t*; *R* thus reflects intrinsic separation efficiency independent of cumulative feed depletion.

Permeate flux, *J* (L m^−2^ h^−1^), was obtained from the cumulative permeate volume collected over a known operating time:

3 *J* = *V*_p_/(*A* × *t*)

where *V*_p_ (L) is cumulative permeate volume, *A* (=3.14 × 10^−4^ m^2^) is the membrane area, and *t* (h) is permeation time; *J* is thus time-averaged, approaching steady state as the membrane wets. Experiments were run in triplicate (*n* = 3); mean ± SD are reported with error bars, and trend ranking/magnitude were preserved across runs. Mass-balance closure was verified for every run before accepting values.

### 2.7. Desorption and regeneration

Following each run, saturated coupons were dismounted and regenerated in methanol, chosen for affinity for thiophenic species and compatibility with PVA/UiO-66. Coupons soaked in methanol (room temp, 4 h) displaced DBT from Zr^4+^ sites and micropores, then were rinsed with *n*-decane and dried under mild vacuum (60 °C) to constant mass.

Regenerated membranes were re-tested under first-cycle baseline conditions (25 °C, 20 bar, 0.5 L min^−1^, 100 mg L^−1^, 120 min) for five cycles. RR (%)—a practical reusability metric, not true regeneration efficiency—was calculated as cycle-*n* capacity over first-cycle capacity (true regeneration efficiency, from methanol-extract DBT recovery, was 89–95%; Section 3.7):

4 RR (%) = *q*_*n*_/*q*_1_ × 100

where *q*_*n*_ (mg g^−1^) is the 120-min sulfur capacity of the membrane in the *n*-th cycle, and *q*_1_ (mg g^−1^) is the corresponding capacity recorded in the first, fully fresh adsorption cycle.

## 3. Results and discussion

### 3.1. Thermogravimetric analysis (TGA)

**Fig. 1:**
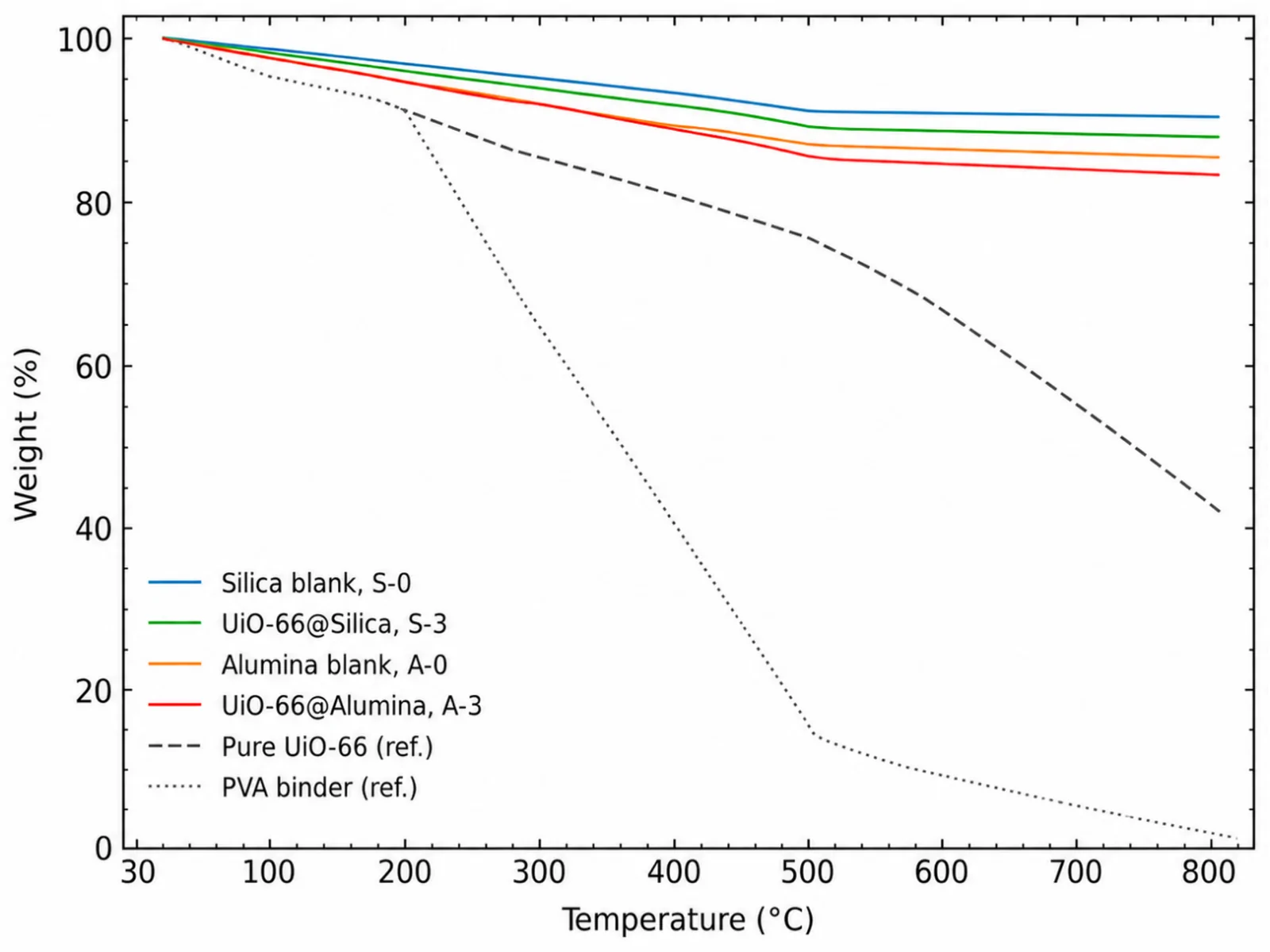
TGA profiles of S-0, A-0, S-3, A-3 (ambient – 800 °C), showing moisture removal, PVA/BDC-linker decomposition, and MOF framework collapse.

The thermal stability of the ceramic supports and UiO-66-loaded composites was assessed from ambient temperature to 800 °C (Fig. 1 and Table 1). Decomposition behavior differed appreciably among the four samples, reflecting the relative contributions of the ceramic backbone, PVA binder, and MOF component.

**Table 1:** Thermogravimetric parameters of the ceramic blanks and UiO-66-modified ceramic composites, including stepwise mass losses across the 30–200, 200–500, and 500–800 °C decomposition windows, total mass loss, and final residue at 800 °C

Sample	Δ*m*_1_ 30–200 °C (%)	Δ*m*_2_ 200–500 °C (%)	Δ*m*_3_ 500–800 °C (%)	Total Δ*m* at 800 °C (%)	Residue at 800 °C (%)
Silica blank, S-0	2.45	6.25	0.85	9.55	90.45
Alumina blank, A-0	3.55	8.00	2.35	13.90	86.10
UiO-66@silica (3%), S-3	2.85	6.95	1.72	11.52	88.48
UiO-66@alumina (3%), A-3	3.85	8.75	2.92	15.52	84.48

For the pristine ceramic blanks, weight loss was shallow overall (Table 1): S-0 showed sequential moisture loss (30–200 °C), PVA-binder degradation (200–500 °C) and a small high-temperature loss, ending at 90.45% residue; A-0 followed the same pattern with slightly larger losses at each step (86.10% residue), consistent with the known thermal stability of amorphous SiO_2_ and crystalline Al_2_O_3_.

UiO-66 incorporation changed the thermograms only modestly (∼3 wt% loading, Table 1): S-3/A-3 each show weak PVA/BDC-linker loss (200–500 °C) and residual collapse (500–800 °C), ending at 88.48%/84.48% residue. Pure UiO-66 loses 58.8% by 800 °C and pure PVA 98.5%, so the small composite increments are consistent with a ∼3 wt% combined MOF + binder contribution.^25,26^

Two points stand out: moisture loss is higher for the MOF-loaded samples (4.85%, S-3; 5.30%, A-3) than the blanks, consistent with UiO-66's known hydrophilicity and extra water on the Zr_6_ cluster, confirmed by FTIR (Section 3.3).^26,27^ Second, composites' residual mass loss after 500 °C contrasts with blanks' near-flat plateau, characteristic of staged linker decomposition/MOF-cluster collapse.^25^ Similar residues (88.48% *vs.* 84.48%) confirm comparable MOF loading.

### 3.2. Textural properties (BET/N_2_ adsorption)

**Fig. 2:**
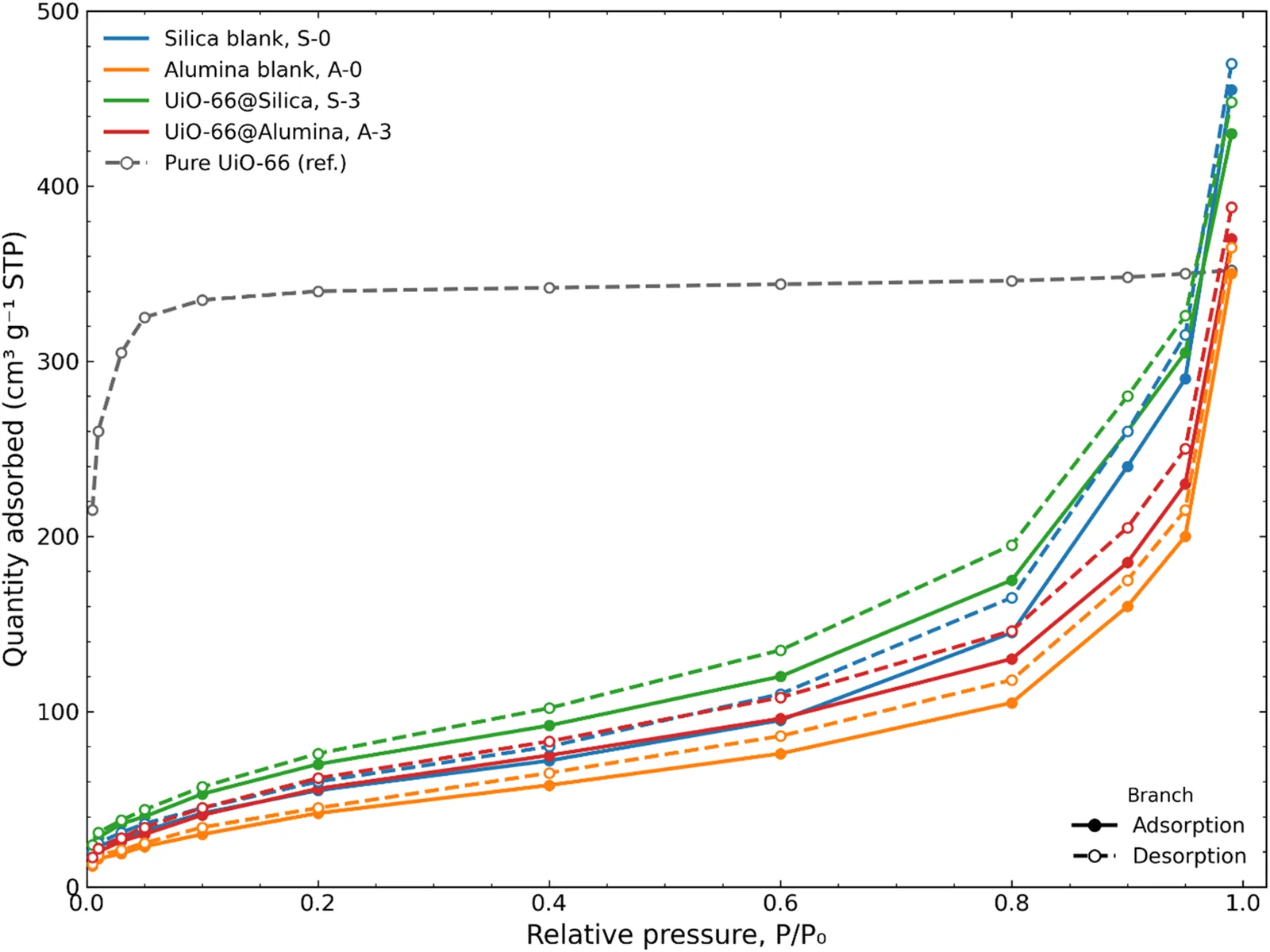
N_2_ adsorption–desorption isotherms of S-0, A-0, S-3, A-3, showing persistent type IV/H2 hysteresis and pore-volume redistribution after MOF incorporation.

N_2_ isotherms (77 K, Fig. 2 and Table 2) are type IV/H2 for all samples, indicating mesoporous networks. S-0 shows a sharp capillary-condensation step (*p*/*p*_0_ = 0.7–0.9, well-developed mesoporosity); A-0 shows lower uptake, consistent with its lower BET surface area/pore volume.

**Table 2:** Textural parameters derived from N_2_ physisorption at 77 K for the ceramic blanks, UiO-66-modified ceramic composites, and pure UiO-66^a^

Sample	*S*_BET_ (m^2^ g^−1^)	*V*_total_ at *p*/*p*_0_ = 0.99 (cm^3^ g^−1^)	*V*_micro (cm^3^ g^−1^)	BJH mean pore diameter (nm)	Isotherm/hysteresis
Silica blank, S-0	300	0.716	0.015	8.4	IV/H2
Alumina blank, A-0	199	0.456	0.012	7.6	IV/H2
UiO-66@silica (3%), S-3	324	0.690	0.025	7.9	IV/H2
UiO-66@alumina (3%), A-3	226	0.462	0.021	7.3	IV/H2
Pure UiO-66	1075	0.52	0.33	1.1	I/IV

a BJH mean mesopore diameter is reported only for the ceramic blanks and UiO-66/ceramic composites because these samples exhibit dominant mesoporosity. For pure UiO-66, the listed value corresponds to the characteristic micropore size of the UiO-66 framework and is therefore not assigned as a BJH-derived mesopore diameter.

S-0 shows the following characteristics: BET 300 m^2^ g^−1^, *V*_total 0.716 cm^3^ g^−1^ (micro 0.015, meso 0.701), BJH 8.4 nm. A-0 is lower on all metrics (199 m^2^ g^−1^; 0.456 cm^3^ g^−1^; 0.012; 0.444; 7.6 nm), from the alumina precursor's lower intrinsic porosity and denser sintered packing.

UiO-66 loading raised BET surface area from 300 → 324 m^2^ g^−1^ (silica, +8%) and 199 → 226 m^2^ g^−1^ (alumina, +14%), consistent with the added porous MOF phase (pure UiO-66: 1075 m^2^ g^−1^; *V*_total 0.52, *V*_micro 0.33 cm^3^ g^−1^), confirming its high surface area and microporous character (XRD/SEM below).

For silica, *V*_total decreased modestly (0.716 → 0.690 cm^3^ g^−1^; mesopore 0.701 → 0.665) while micropore volume rose (0.015 → 0.025), consistent with partial mesopore filling by UiO-66 plus new MOF micropores. For alumina, *V*_total was essentially unchanged (0.456 → 0.462 cm^3^ g^−1^) while micropore volume still rose (0.012 → 0.021), suggesting that UiO-66 sits mainly at the external surface/mesopore entrances without occluding the network. BJH diameter decreased slightly for both (8.4 → 7.9 nm silica; 7.6 → 7.3 nm alumina). Pure UiO-66's pore size is reported separately, being predominantly microporous.

Sulfur-binding sites per unit UiO-66 area can be estimated from Table 4 data: the MOF-attributable increment is ≈100 (S-3) and ≈96 (A-3) mg S per g UiO-66. Referenced to pure UiO-66's BET surface area (1075 m^2^ g^−1^), this gives ≈1.7–1.8 S atoms per nm^2^ for both—a close match supporting a common, MOF-intrinsic mechanism and confirming that the uptake is consistent with the ICP-confirmed loading.

This external siting alone does not make A-3 higher-capacity than S-3, since total sites track total surface area/micropore volume, both lower for alumina (BET 199 → 226 *vs.* 300 → 324 m^2^ g^−1^; *V*_micro 0.021 *vs.* 0.025 cm^3^ g^−1^). As per-gram-UiO-66 capacities are nearly identical, A-3's lower absolute capacity reflects its smaller area/micropore volume, not lower per-site accessibility.

The persistence of type IV isotherms with H2-type hysteresis in both composites confirms that the mesoporous nature of the ceramic hosts is largely retained after MOF incorporation.^28^ The coexistence of preserved meso-transport channels and the Zr-rich UiO-66 phase is advantageous: mesopores facilitate mass transport while UiO-66 provides high-affinity adsorption sites for thiophenic sulfur.^17,29^

### 3.3. FTIR spectral analysis

**Fig. 3:**
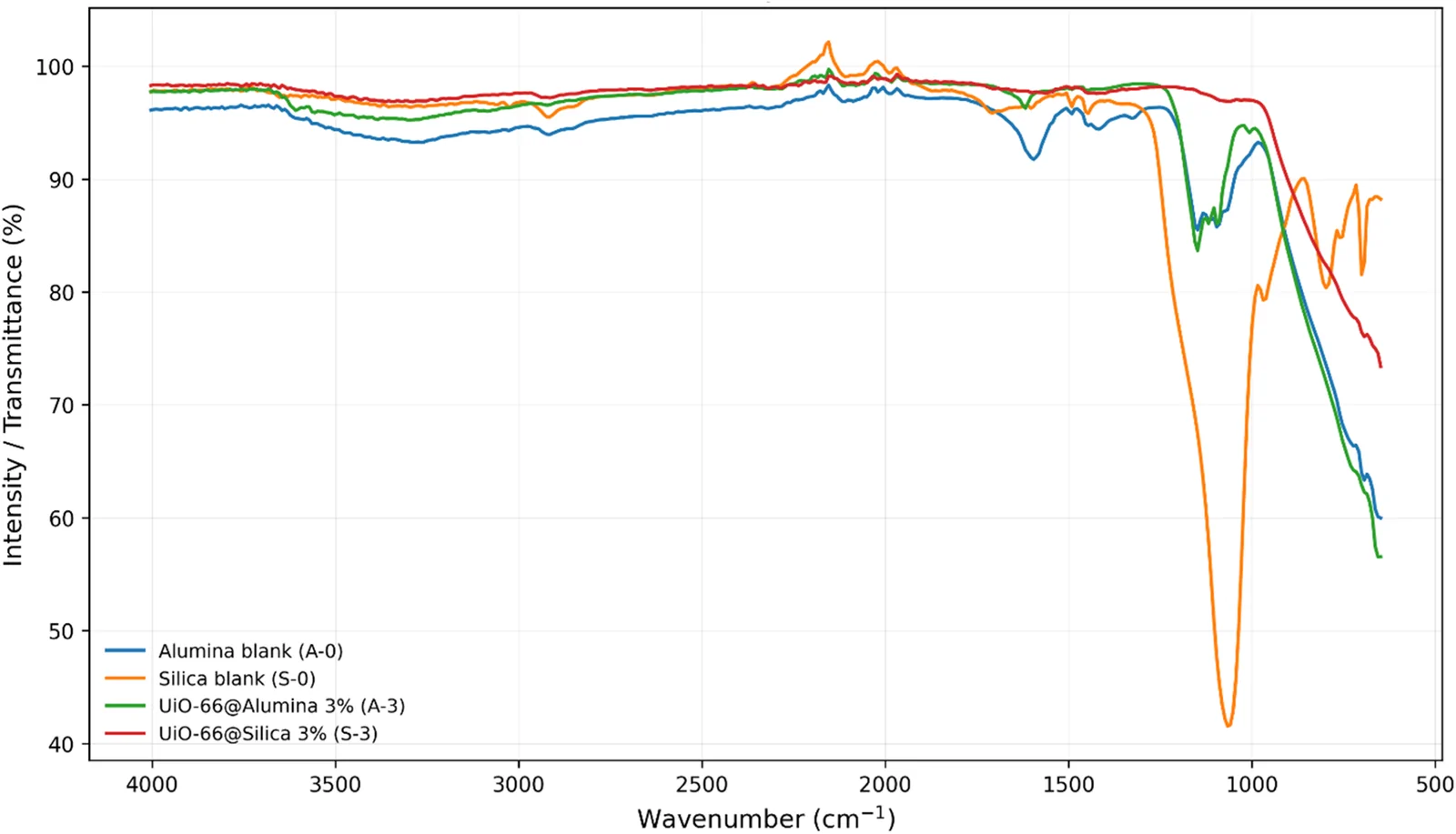
FTIR spectra of S-0, A-0, S-3, and A-3, showing ceramic-framework absorptions (Si–O–Si, Al–O) alongside carboxylate and Zr–O signatures from embedded UiO-66.

Fig. 3 compares FTIR transmittance spectra of the four samples (4000–500 cm^−1^). The ceramic blanks show typical oxide vibration patterns: S-0 exhibits O–H stretching (3440 cm^−1^) and Si–O–Si asymmetric/symmetric stretching (1085/803 cm^−1^) with Si–OH bending; A-0 shows O–H stretching (3435 cm^−1^), Al–O lattice vibration (760 cm^−1^), AlO_6_/AlO_4_ network vibrations below 900 cm^−1^, and a 1600 cm^−1^ band from physisorbed-water bending.

After UiO-66 incorporation, the ceramic skeletal vibrations persist, but new MOF-diagnostic peaks appear: asymmetric/symmetric carboxylate (–COO–) stretching at 1585/1395 cm^−1^ (S-3) and 1583/1392 cm^−1^ (A-3) from the deprotonated BDC linker coordinated to Zr_6_ clusters, and a new Zr–O/Zr–O–C band at 665/663 cm^−1^. The 3400 cm^−1^ hydroxyl envelope is broader in composites, consistent with µ_3_-OH on Zr_6_O_4_(OH)_4_ nodes and higher adsorbed water (TGA, Section 3.1).

A subtle broadening and a slight redshift of the dominant ceramic bands are visible after MOF loading, which can be rationalized by interfacial interactions between the carboxylate/Zr–OH groups of UiO-66 and the surface –OH groups of silica or alumina.^26,30^ These features support successful UiO-66 integration into both matrices without disrupting core framework vibrations.^31^

### 3.4. X-ray diffraction (XRD) analysis

**Fig. 4:**
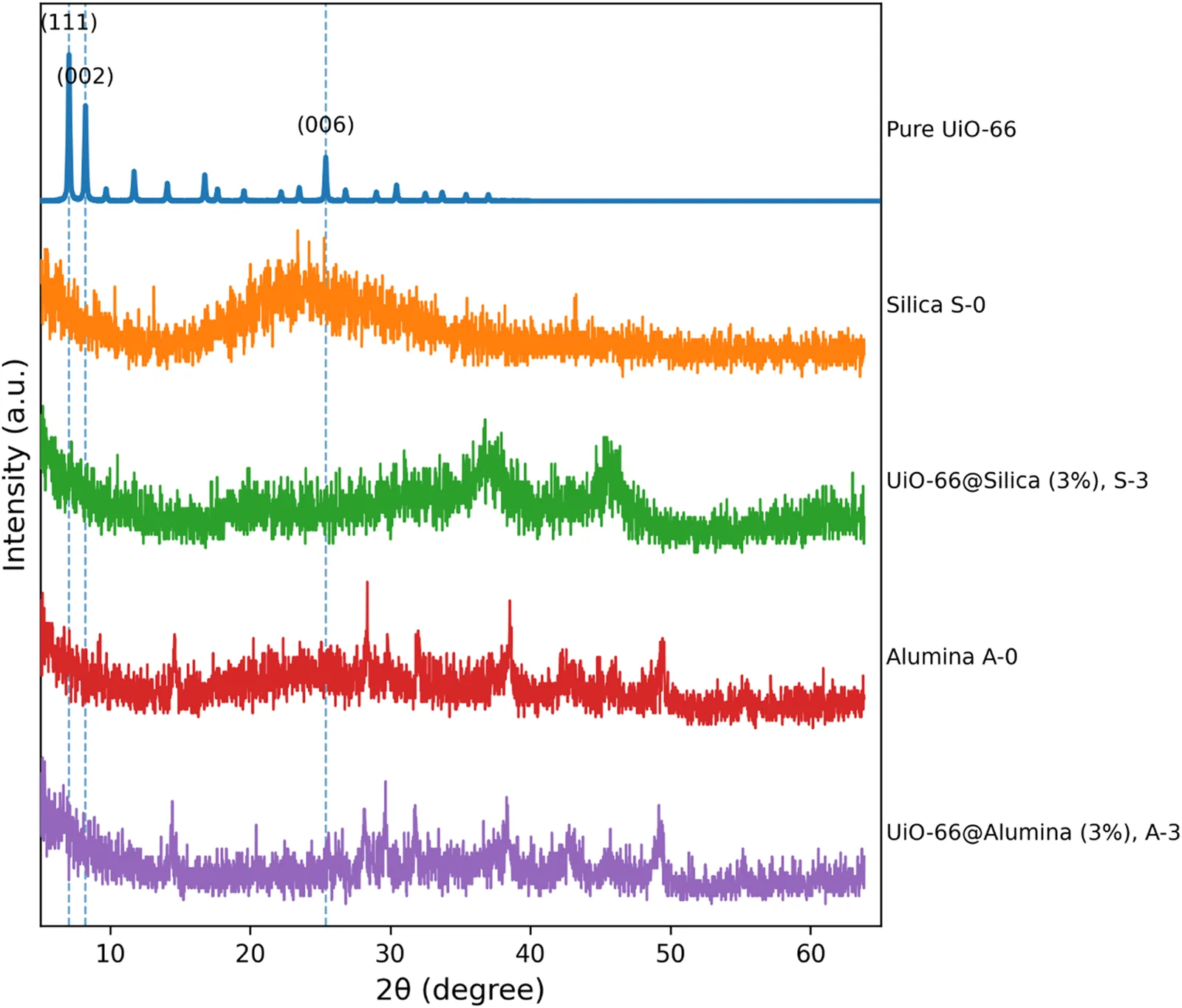
Stacked XRD patterns (Cu Kα, *λ* = 1.5406 Å; 2*θ* = 4–65°) of pure UiO-66, S-0, S-3, A-0, and A-3. Dashed lines mark the expected positions of the three diagnostic UiO-66 reflections (111, 002, 006), unresolved at 3 wt% loading above the amorphous ceramic background; chemical evidence for incorporation comes from SEM, N_2_ physisorption, FTIR, and ICP-OES (Sections 3.2, 3.3 and 3.8).

Powder XRD patterns of the four samples, together with the pure UiO-66 pattern are shown as a stacked plot in Fig. 4.

S-0 displays a diffuse, amorphous pattern with a broad hump near 2*θ* ≈ 22° from disordered SiO_2_/short-range Si–O–Si ordering, while A-0 shows a moderately crystalline pattern of broadened reflections on a sloping background, consistent with a transitional alumina phase of limited long-range order.

The pure UiO-66 reference pattern (top panel, Fig. 4) clearly shows the three diagnostic reflections at 2*θ* = 7.06° (111), 8.23° (002), and 25.39° (006), confirming the face-centered cubic *Fm̄m* lattice.^25^

At 3 wt%, UiO-66 reflections are not resolved above the ceramic background in S-3/A-3 (expected positions marked by dashed lines, Fig. 4), which is expected, since diffracted MOF intensity falls at/below detection at such loadings, widely reported for MOF/ceramic composites.^26,31^ Since XRD cannot resolve the MOF phase, incorporation is established *via* four complementary techniques below; XPS was not performed but is noted as future work. Host signatures (silica's amorphous hump in S-3; alumina's broadened reflections in A-3) are preserved, confirming that the matrix is undamaged.

Crystalline UiO-66 incorporation at 3 wt% is confirmed by four techniques: SEM (uniform Zr, ≈1 wt%, RSD ≈8%, Section 3.8); N_2_ physisorption (micropore volume 0.015 → 0.025 cm^3^ g^−1^, S-0 → S-3, Section 3.2); FTIR (new bands at 1585, 1395, and 665 cm^−1^, Section 3.3); and ICP-OES (2.98/2.91 wt% Zr-derived loading, Section 3.8). Together these confirm successful crystalline integration with each support intact.

### 3.5. Morphological analysis (SEM)

**Fig. 5:**
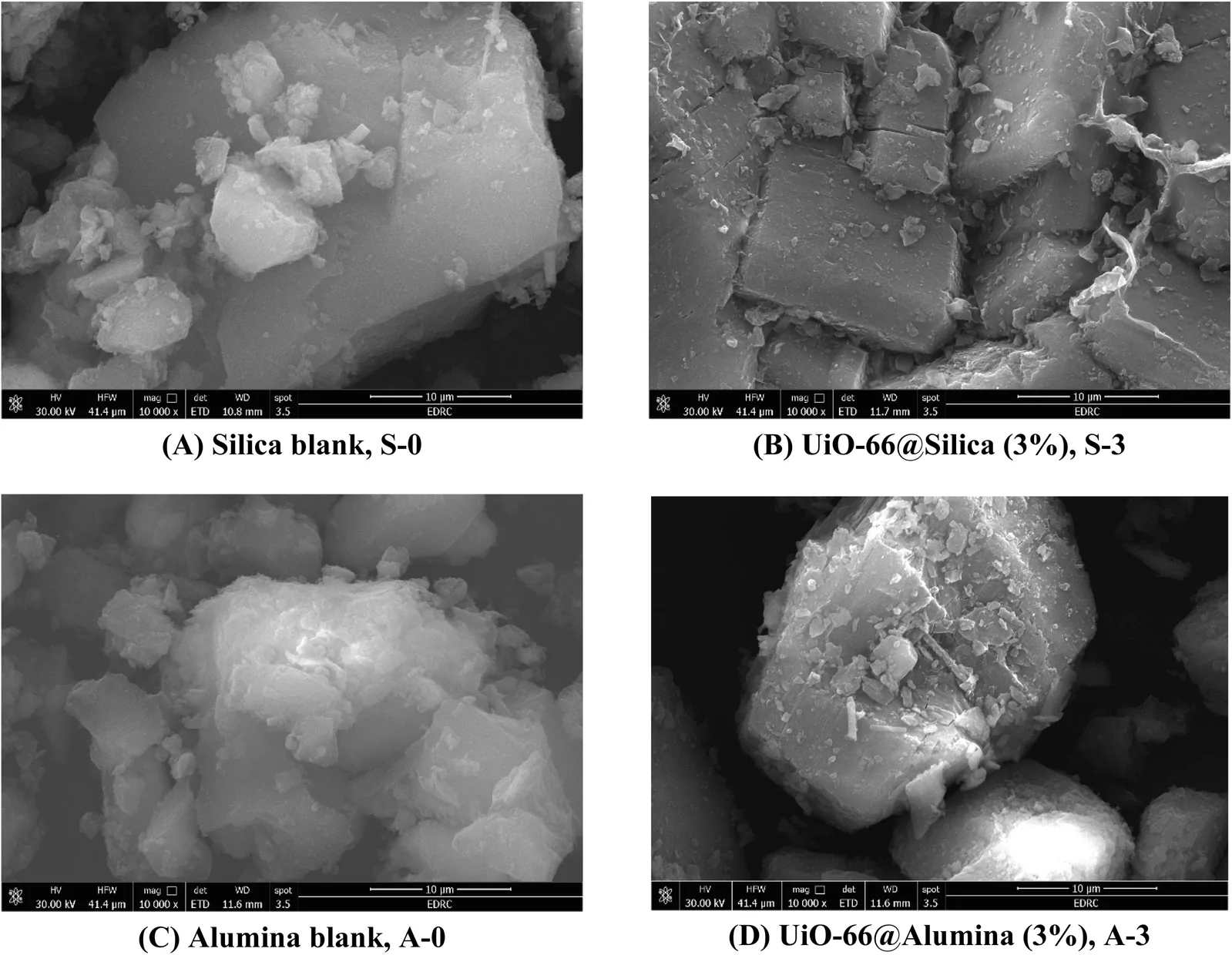
SEM micrographs of (A) silica blank S-0, (B) UiO-66@silica S-3, (C) alumina blank A-0, and (D) UiO-66@alumina A-3, all acquired at the same magnification (100 00×) to allow direct comparison of surface morphology before and after UiO-66 incorporation.

Fig. 5 presents SEM micrographs of the unmodified and UiO-66-modified ceramic supports acquired at the same magnification (100 00×), enabling direct comparison of their surface morphologies before and after MOF incorporation. The silica blank (Fig. 5A) exhibits relatively smooth particle surfaces with distinct grain boundaries and large compact particles. Only limited surface irregularities and a small number of fine particulates are observed, indicating a comparatively uniform morphology. In contrast, the alumina blank (Fig. 5C) displays a rougher and more heterogeneous texture, characterized by fractured particles, irregular edges, and flake-like surface features, reflecting the intrinsically more textured nature of alumina-based materials.

Following the incorporation of 3 wt% UiO-66, noticeable changes in surface morphology are evident. The UiO-66@silica composite (S-3, Fig. 5B) exhibits an increase in surface roughness accompanied by the appearance of numerous fine particles dispersed across the support surface. Small deposits can be observed on particle faces, edges, and interparticle regions, producing a more textured morphology than that of the unmodified silica support. Similarly, the UiO-66@alumina composite (A-3, Fig. 5D) shows a higher density of attached particulates and plate-like fragments distributed over the alumina grains. Compared with the alumina blank, the surface appears more extensively decorated and roughened, suggesting the presence of an additional dispersed phase on the support surface.

The observed morphological changes are consistent with successful incorporation of UiO-66 within both ceramic matrices. Rather than forming a continuous coating layer, the deposited phase appears as finely dispersed surface-associated particles distributed throughout the support grains. This behavior agrees with previous reports describing UiO-66-based materials, where the MOF phase is commonly observed as dispersed particulate deposits contributing to increased surface texture and heterogeneity.^30–33^ The effect is particularly pronounced for the alumina-based composite, where the density of surface deposits and degree of surface roughening are greater than those observed for the silica-based sample.

No visible powder shedding, cracking, or particulate loss was observed during handling of any coupon throughout preparation, mounting, and post-run recovery, consistent with the coupon-level dimensional and mass stability reported in Section 3.8 (≈97% flux retention, <0.4% mass change, <0.6% thickness change after operation). Although SEM alone cannot conclusively identify the deposited particles as UiO-66, the clear morphological differences between the blank and modified samples provide evidence of successful surface modification. Furthermore, these observations are consistent with the structural and textural characterization results obtained from XRD, FTIR, and BET analyses. The increased surface roughness and additional surface-associated particles introduced after UiO-66 loading are expected to contribute additional accessible adsorption sites while maintaining the integrity of the ceramic support. Collectively, the characterization results indicate effective incorporation of UiO-66 into both silica- and alumina-based ceramics, generating hierarchical porous composites that are favorable for adsorptive desulfurization applications.^30–33^

### 3.6. Membrane desulfurization performance

The loading-screening series was first evaluated under the baseline cross-flow conditions using the DBT/*n*-decane model fuel (Section 2.5). Adsorption capacity and sulfur rejection were considered together to select the UiO-66 loading. The effects of pressure, concentration, and flow rate were then examined using the blanks and the selected 3 wt% formulations, and the results were interpreted in relation to the structural evidence in Sections 3.1–3.5.

#### 3.6.1. Effect of UiO-66 loading and selection of the 3 wt% formulation

**Fig. 6:**
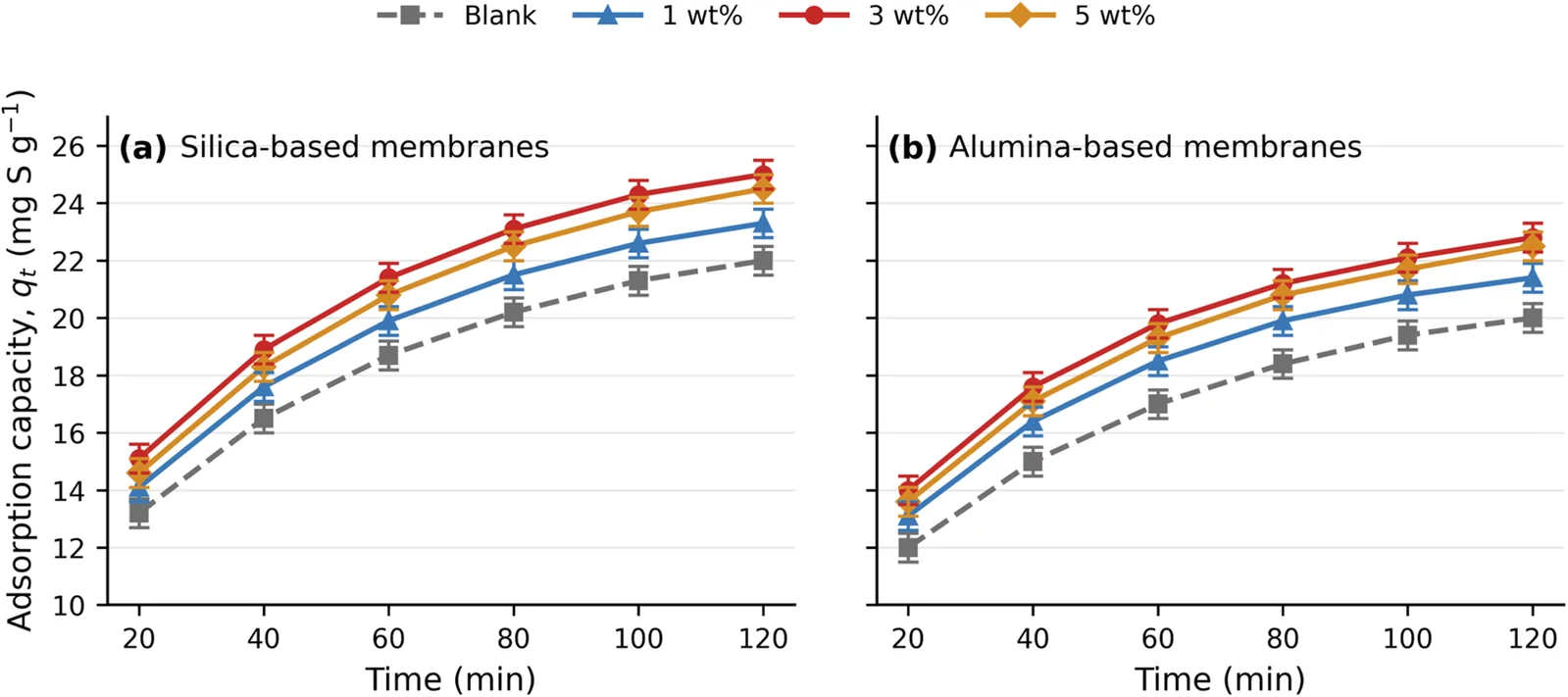
Effect of nominal UiO-66 loading on the time-dependent sulfur adsorption capacity of (a) silica-based and (b) alumina-based PVA-bound ceramic-composite membranes at 25 °C, 20 bar, 0.5 L min^−1^, and 100 mg S per L. Values are presented as mean ± SD (*n* = 3).

Before investigating the operating parameters, the influence of nominal UiO-66 loading was evaluated at 1, 3, and 5 wt% under the baseline conditions of 25 °C, 20 bar, 0.5 L min^−1^, and 100 mg S per L; the corresponding blank membranes were included as references. As shown in Fig. 6, adsorption capacity increased continuously with time for every formulation. None of the curves reached a clearly defined equilibrium plateau within 120 min; consequently, the reported values represent time-dependent sulfur uptake rather than equilibrium adsorption capacity.

At 120 min, the silica series reached 22.0, 23.3, 25.0, and 24.5 mg S per g for S-0, S-1, S-3, and S-5, respectively, whereas the alumina series reached 20.0, 21.4, 22.8, and 22.5 mg S per g for A-0, A-1, A-3, and A-5. Thus, the 3 wt% formulations increased the 120-min capacity by 13.6% for silica and 14.0% for alumina relative to their corresponding blanks, whereas increasing the loading to 5 wt% produced no further improvement. The higher uptake after UiO-66 incorporation is consistent with (i) physisorption in sub-nanometer cavities (approximately 6 Å windows and 8/11 Å cages), matched to DBT (approximately 9 × 7 × 4 Å), and (ii) Lewis acid–base coordination between the DBT sulfur lone pair and unsaturated Zr^4+^ sites, including missing-linker defects,^34^ and (iii) π–π/dispersive interactions between BDC and DBT's aromatic rings.^17,18^ These mechanisms are consistent with the BET and SEM evidence in Sections 3.2 and 3.5.

**Fig. 7:**
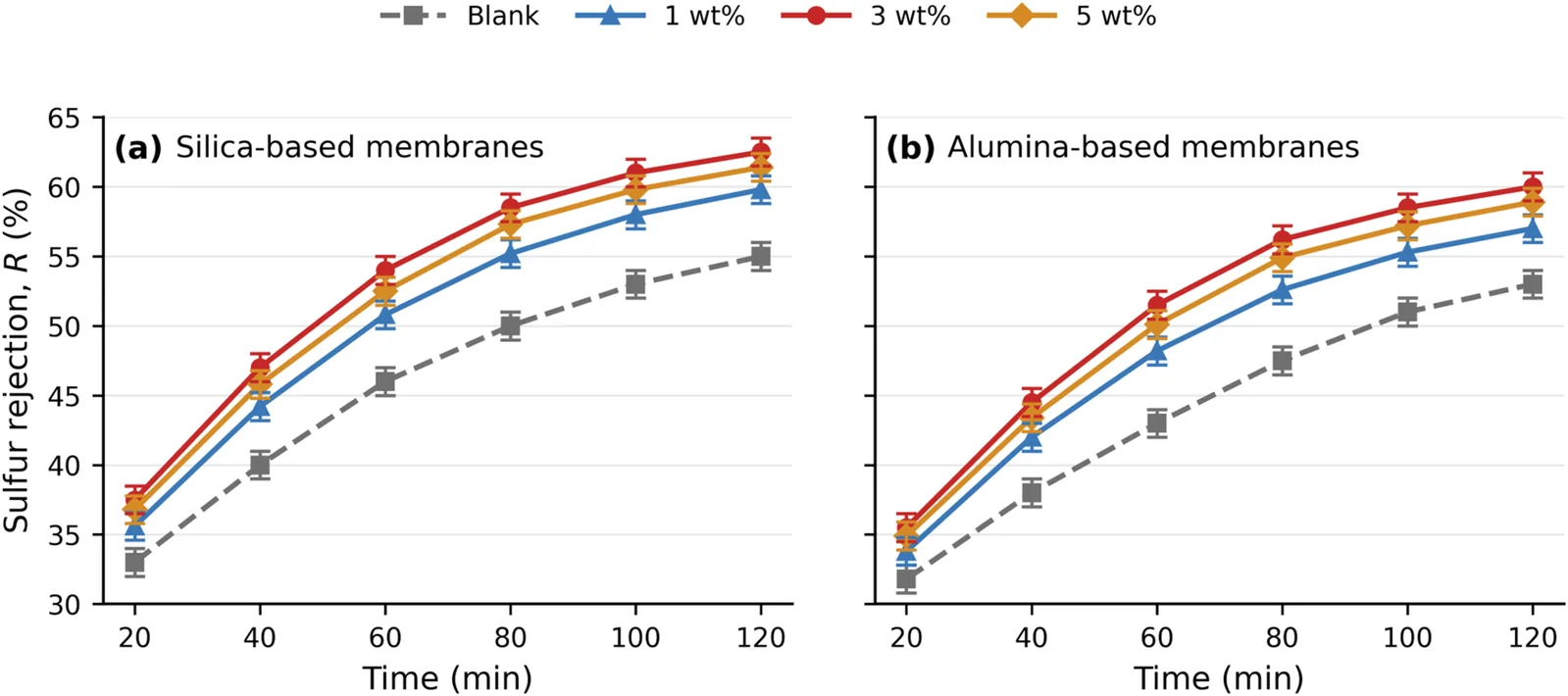
Effect of nominal UiO-66 loading on the time-dependent sulfur rejection of (a) silica-based and (b) alumina-based PVA-bound ceramic-composite membranes at 25 °C, 20 bar, 0.5 L min^−1^, and 100 mg S per L. Values are presented as mean ± SD (*n* = 3).

The silica-based formulations consistently exhibited higher capacities than the corresponding alumina-based formulations, in agreement with the higher BET surface area and pore volume of the silica support. Within the selected formulations, the higher capacity of S-3 relative to A-3 likewise agrees with its greater BET surface area (324 *vs.* 226 m^2^ g^−1^). The blank-normalized capacity increments correspond to approximately 100 and 96 mg S per gram of UiO-66 for S-3 and A-3, respectively (Table 4). Because UiO-66 is only 3 wt% of the composite, the 25 mg S per g capacity of S-3 can equivalently be partitioned by mass fraction: the 97 wt% ceramic backbone accounts for ≈22 mg S per g (88% of the total, matching S-0's blank capacity), while the 3 wt% UiO-66 accounts for the remaining ≈3 mg S per g (12% of the total). The two views are complementary rather than contradictory: on a whole-composite basis the ceramic backbone supplies most of the absolute uptake simply because it is 97% of the mass, whereas on a per-gram basis UiO-66 is roughly 4.4-fold more active than the ceramic matrix (≈100 *vs.* ≈22.7 mg S per g ceramic). We attribute this disproportionate per-gram efficiency to the additional Zr^4+^ Lewis-acid sites and BDC π-systems that UiO-66 introduces—a chemical environment absent from the unmodified ceramics—so that although the 3 wt% MOF increment adds only ≈3 mg S per g in absolute terms, it accounts for the full 12% capacity gain over the blank, together with the larger relative gains in rejection and cycling retention (Section 3.7). A bounded raw-material cost estimate places this efficiency gain in economic context: using list prices for ZrCl_4_ (∼$2.00 per g) and H_2_BDC (∼$0.53 per g) plus typical bulk prices for the ceramic, PVA, and solvents, UiO-66 costs an estimated $4.80–5.70 per g and raises the composite's raw-material cost by ∼13% over the blank (≈$1.10 *vs.* $0.98 per g of membrane). This is essentially cost-neutral per mg S captured on a single 120-min pass (≈$0.044 per mg S for both formulations); over the five-cycle reuse lifetime (Table 3), however, S-3's better capacity retention lowers its cost to ≈$0.0094 per mg S *versus* ≈$0.0103 per mg S for S-0—roughly 8% cheaper per mg S captured. This estimate covers raw materials only, excluding labor, energy, and equipment, and should be read as an order-of-magnitude comparison rather than a formal techno-economic analysis, identified here as a priority for future work ahead of any pilot- or industrial-scale evaluation. Because the present configuration is a continuous, recirculating cross-flow system rather than a fixed-bed packed column, conventional breakthrough curves—which track a moving concentration front along a bed length—are not directly applicable to this membrane geometry; the time-resolved capacity and rejection data of Fig. 6 and 7 are the corresponding metrics for this configuration. Based on the 120-min rejection values, the estimated permeate concentrations [*C*_0_(1 − *R*)] were approximately 45 and 47 mg S per L for S-0 and A-0 and 37.5 and 40 mg S per L for S-3 and A-3, respectively.

**Table 3:** Summary of the desulfurization performance of the four ceramic membranes at the baseline operating conditions (25 °C, 20 bar, 0.5 L min^−1^, 100 mg L^−1^) at 120 min. Values for adsorption capacity, sulfur rejection, and permeate flux are taken at the 120-min sampling point

Sample	Capacity at 120 min (mg g^−1^)	Rejection at 120 min (%)	Flux at 120 min (L m^−2^ h^−1^)	Capacity retention after 5 cycles (%)
Silica blank, S-0	22.0	55.0	175	73
Alumina blank, A-0	20.0	53.0	168	70
UiO-66@silica (3%), S-3	25.0	62.5	157.5	88
UiO-66@alumina (3%), A-3	22.8	60.0	153	85

Beyond its adsorptive contribution, UiO-66 loading measurably alters the pore architecture of both ceramic hosts: BJH mean pore diameter narrows (8.4 → 7.9 nm for silica; 7.6 → 7.3 nm for alumina) and micropore volume rises (Section 3.2), while SEM shows the framework distributed at grain boundaries and interparticle regions rather than as a continuous overlayer (Section 3.5). This altered pore geometry and interchannel connectivity is a membrane-level behavior not accessible to either the pristine ceramic or the free MOF powder, and is a preliminary indication that the internal transport architecture of ceramic membranes—an aspect largely unexploited in fuel-treatment membranes to date—can be deliberately reshaped through MOF loading. This suggests a route toward introducing additional functional groups on the UiO-66 linker for tailored sulfur selectivity in future composite generations.^26,30,31^

The loading-dependent sulfur-rejection results showed the same non-monotonic response (Fig. 7). At 120 min, the silica-based series reached 55.0, 59.8, 62.5, and 61.4% for S-0, S-1, S-3, and S-5, respectively, while the alumina-based series reached 53.0, 57.0, 60.0, and 58.9% for A-0, A-1, A-3, and A-5. At 20 min, S-3 and A-3 gave rejection values of 37.5 and 35.5%, respectively. Relative to the corresponding blanks, the 3 wt% formulations increased 120-min rejection by 7.5 percentage points for silica and 7.0 percentage points for alumina. The 3 wt% formulation was numerically superior to the 1 and 5 wt% formulations at every sampling time for both supports.

For the blank ceramic supports, sulfur removal is dominated by non-specific physisorption within the type IV/H2 mesopores, with only weak size-exclusion effects. The greater rejection after UiO-66 incorporation is consistent with high-affinity adsorption in the MOF micropores through Lewis acid–base coordination to Zr^4+^ centers, π–π interactions with the BDC linkers, and confinement that increases DBT residence time near active sites.^17,23,29^ The combined adsorption and transport response is consistent with the established permeability–selectivity trade-off.^24^ The slight decline at 5 wt% may reflect less favorable UiO-66 dispersion, partial obstruction of ceramic transport pathways, or a smaller fraction of accessible added sites; loading-specific morphological and textural measurements would be required to distinguish conclusively among these possibilities.

The absence of any further improvement at 5 wt% shows that performance was not directly proportional to the amount of UiO-66 incorporated. Because the 3 wt% formulations simultaneously provided the highest measured adsorption capacity and sulfur rejection within the investigated range, 3 wt% was selected for the subsequent operating-parameter studies. Accordingly, S-3 and A-3 were evaluated alongside their corresponding blanks in the pressure, concentration, flow-rate, regeneration, and stability investigations. This non-monotonic response—improvement from a blank up to a low-to-moderate MOF loading, followed by a plateau or slight decline at higher loading—is consistent with the broader composite-membrane literature, where the very high intrinsic surface area and microporosity of frameworks such as UiO-66 means that even low (1–5 wt%) loadings can be adsorption-effective, while higher loadings increasingly promote particle agglomeration and partial pore blockage that offset the benefit of the added active-site density.^17–19,29^ A single-digit wt% loading is therefore also the more economical choice, since it captures most of the attainable performance gain without a proportional increase in MOF content and cost (Section 3.6.1).

#### 3.6.2. Effect of pressure

**Fig. 8:**
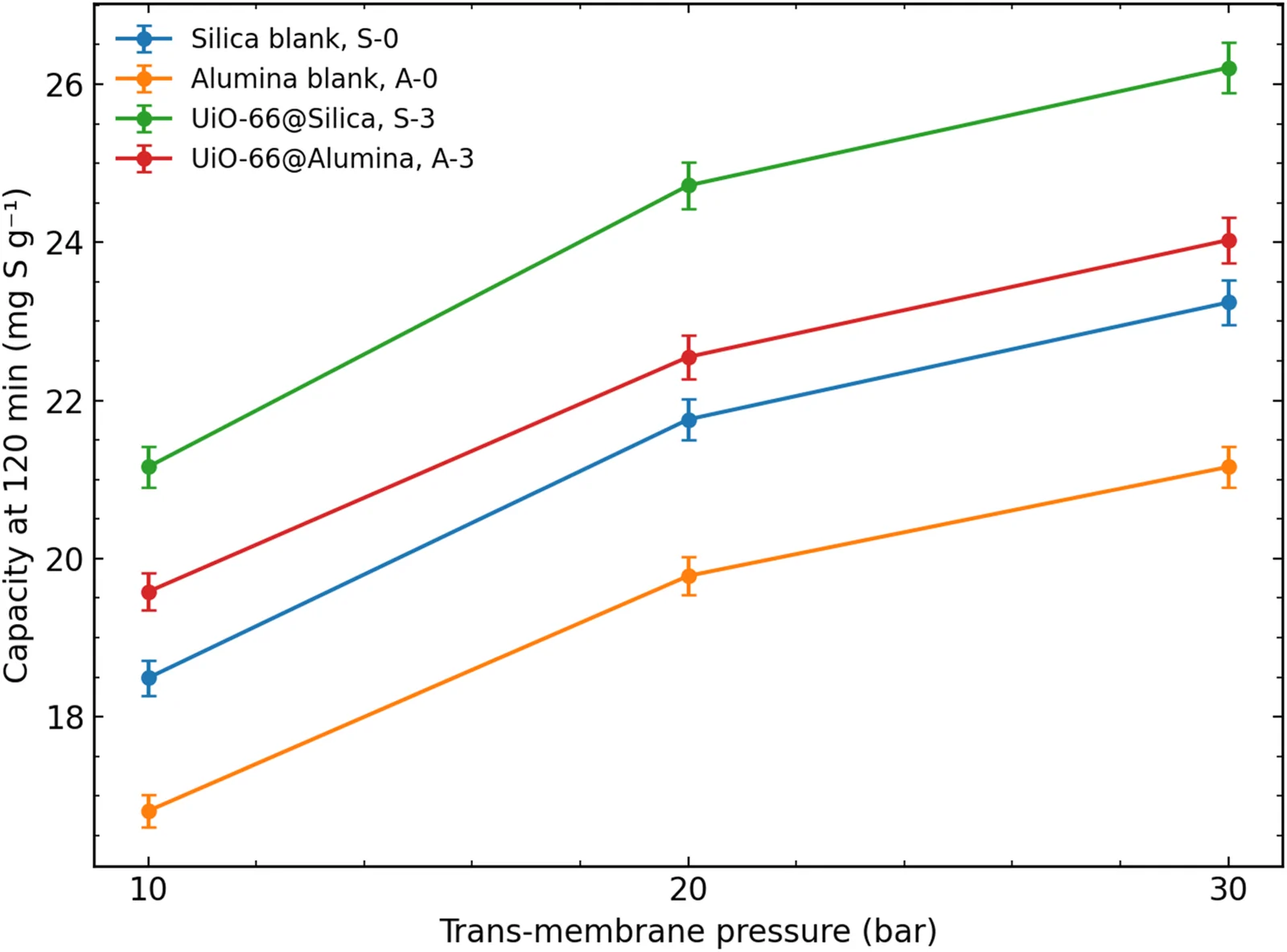
Effect of pressure (10, 20, 30 bar) on 120-min capacity (25 °C, 0.5 L min^−1^, 100 mg L^−1^) for (a) S-0, (b) A-0, (c) S-3, (d) A-3.

The effect of *trans*-membrane pressure (10, 20, 30 bar) on capacity at 25 °C, 0.5 L min^−1^, 100 mg L^−1^ is shown in Fig. 8. Blanks rise modestly (S-0: 18.5 → 23.2; A-0: 16.8 → 21.2 mg g^−1^, 120 min). Composites show a comparable response at slightly higher capacities (S-3: 21.2 → 26.2; A-3: 19.6 → 24.0 mg g^−1^).

#### 3.6.3. Effect of concentration

**Fig. 9:**
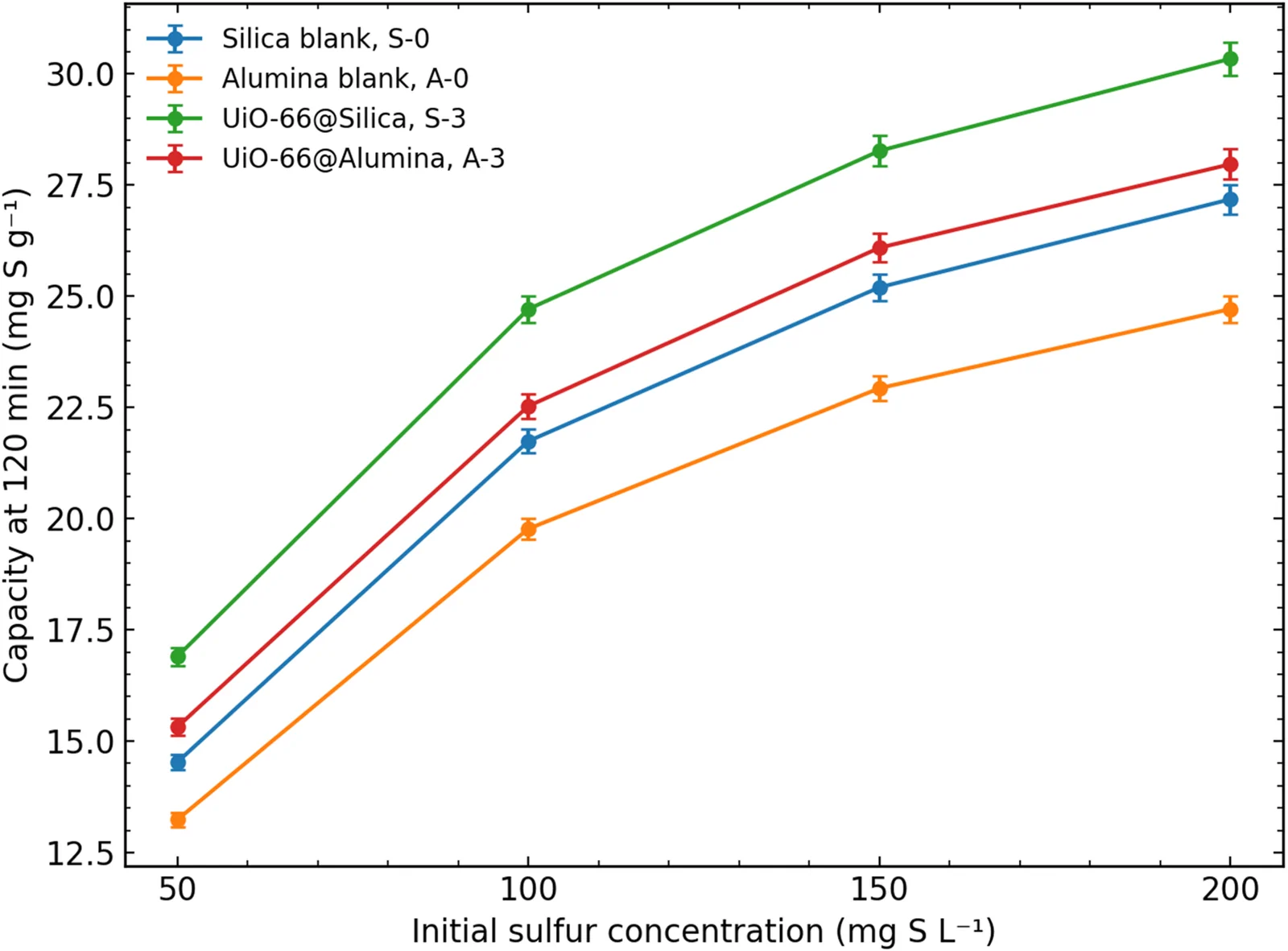
Effect of concentration (50–200 mg L^−1^) on 120-min capacity (25 °C, 20 bar, 0.5 L min^−1^) for (a) S-0, (b) A-0, (c) S-3, and (d) A-3.

The influence of feed concentration (50–200 mg L^−1^) on capacity at 25 °C, 20 bar, 0.5 L min^−1^ is shown in Fig. 9. Uptake at 120 min grows monotonically with concentration for all samples, from a stronger mass-transfer gradient. Silica blank: 14.5 → 27.2 mg g^−1^; alumina blank: 13.2 → 24.7 mg g^−1^ (50 → 200 mg L^−1^). Composites run above their blanks with a comparable slope but higher intercept: S-3 16.9 → 30.3; A-3 15.3 → 28.0 mg g^−1^.

Two trends are apparent. First, the capacity increments between consecutive concentration levels shrink at higher concentrations, especially for the composites, as expected for adsorption-controlled uptake rather than pure linear partitioning,^17,22^ reflecting progressive saturation of high-affinity Zr sites. Second, the capacity advantage of S-3 and A-3 over their blanks was maintained at all investigated concentrations.

#### 3.6.4. Effect of flow rate

**Fig. 10:**
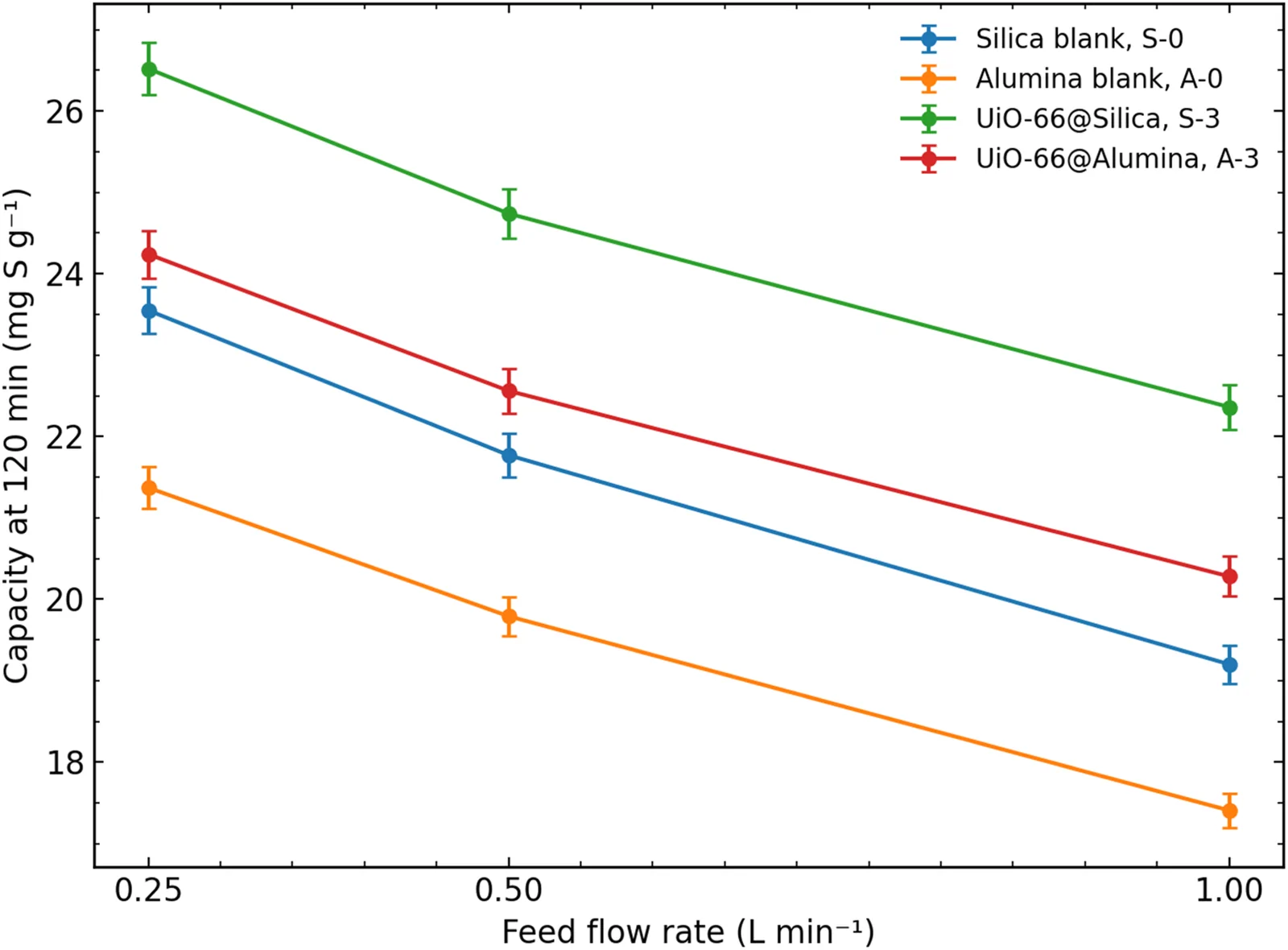
Effect of flow rate (0.25–1.0 L min^−1^) on 120-min capacity (25 °C, 20 bar, 100 mg L^−1^) for (a) S-0, (b) A-0, (c) S-3, and (d) A-3.

Fig. 10 shows flow-rate effects (0.25–1.0 L min^−1^) on capacity (25 °C, 20 bar, 100 mg L^−1^): highest capacities at the lowest rate for all samples (S-0/A-0: 23.6 → 19.2/21.4 → 17.4; S-3/A-3: 26.5 → 22.4/24.2 → 20.3 mg g^−1^).

This fits residence-time-limited adsorption: low flow rate allows more complete diffusion and site access, while higher flow rate shortens contact time so more sulfur bypasses long-range diffusion into the meso-/micropores.^35^ The throughput/removal-efficiency trade-off (Fig. 10), typical of adsorption membranes, points to an intermediate operating point.^36^ The loading screen in Section 3.6.1 demonstrated diminishing returns at 5 wt% under baseline conditions; whether the same loading ranking is retained at the highest flow rate requires a dedicated loading–flow interaction study.

### 3.7. Regeneration and cycling stability

**Fig. 11:**
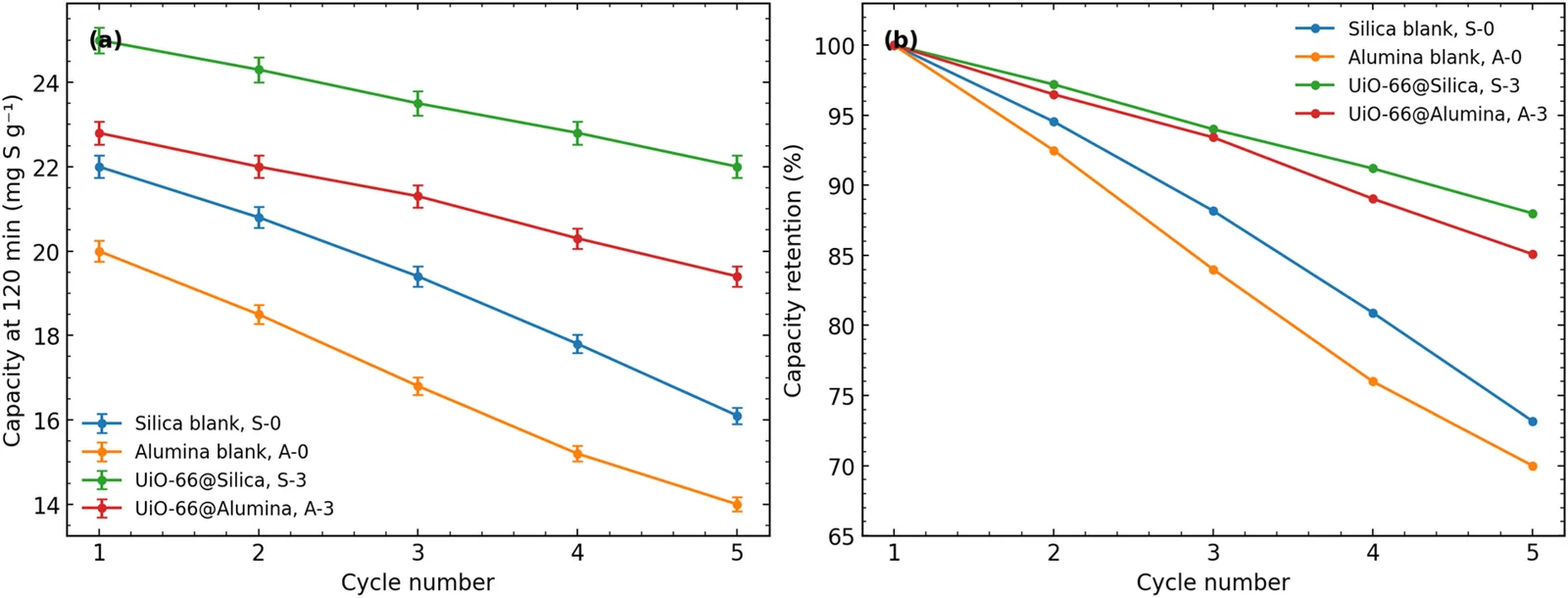
Cycling stability of S-0, A-0, S-3, and A-3 over five adsorption–regeneration cycles (25 °C, 20 bar, 0.5 L min^−1^, 100 mg L^−1^, 120 min): (a) absolute capacity per cycle; (b) capacity-retention ratio (RR, %) *vs.* cycle 1.

Reusability was probed over five adsorption–regeneration cycles using the methanol protocol of Section 2.7. The 120-min capacity in each cycle and the capacity–retention ratio (RR, %, eqn (4)) are shown in Fig. 11a and b.

All four samples show a monotonic capacity decline, but far more steeply for the blanks: RR falls from 100% (cycle 1) to 73% (S-0) and 70% (A-0) by cycle 5, *versus* 88% (S-3) and 85% (A-3) for the UiO-66-loaded composites—a 12–15% loss over five reuses.

Chemically, blank uptake reflects non-specific DBT physisorption on silanol/aluminol sites; methanol displaces some DBT competitively, but tightly bound species (dimers, oxidation by-products) survive the soak and block sites over cycles. In UiO-66 composites, Lewis acid–base coordination to Zr^4+^ centers and π–π/confinement interactions within the sub-nanometer cavities are the predominant adsorption modes; methanol, a polar protic solvent, displaces DBT from these sites each cycle, and the rigid framework presents similar site density next cycle.^17,26^ S-3's slightly higher retention *vs.* A-3 fits its larger BET surface area (Section 3.2) and discrete, well-anchored cauliflower-like aggregates (SEM, Section 3.5), apparently less detachment-prone than alumina's uniform sub-micron decoration.^31^

### 3.8. Loading verification, competitive adsorption, and stability

Composition and robustness were probed further. ICP-OES gave UiO-66 loadings of 2.98/2.91 wt% (S-3/A-3); SEM showed uniform Zr at ≈1 wt% (RSD ≈ 8%), agreeing with the nominal 3 wt%. A single-pass sulfur mass balance closed to ≈100% of the 25 mg charged. The simplified DBT/*n*-decane system isolates formulation-variable effects, providing the mechanistic baseline for real-fuel matrices, with S-3 prioritized for future refinery studies. Stability testing showed only minor framework loss: Zr leaching 8.6/10.4 µg L^−1^ (S-3/A-3), PVA/organic carbon ≤0.5 mg L^−1^, and coupons retained ≈97% of solvent flux with <0.4% mass/<0.6% thickness change, consistent with cycling-inferred framework integrity.

Expressed as a fraction of total framework Zr, leaching was small. Using the measured cumulative permeate volumes over 120 min (approximately 98.9 mL for S-3 and 96.1 mL for A-3), the corresponding Zr losses were approximately 0.85 and 1.0 µg. Based on the ICP-confirmed UiO-66 loadings and the stoichiometric Zr fraction of UiO-66 [Zr_6_O_4_(OH)_4_(BDC)_6_, approximately 33 wt%], the framework Zr contents per coupon were approximately 2.45 and 2.39 mg for S-3 and A-3, giving fractional losses of approximately 0.03 and 0.04%, respectively. This remains an idealized stoichiometric estimate rather than an independent framework mass balance.

Table 4 places these results in context against representative UiO-66-based desulfurization systems. Most prior UiO-66 work uses the framework as a powder in batch adsorption.^23,37,38^ The only reported UiO-66 membrane for desulfurization is a tubular UiO-66-NH_2_ polycrystalline membrane operating by pervaporation—a fundamentally different, vapor-pressure-driven mechanism.^39^ The present cross-flow membranes are thus a distinct configuration: continuous, pressure-driven adsorptive filtration combining UiO-66's capacity with ceramic mechanical robustness in one body.

**Table 4:** Comparison of representative UiO-66-based desulfurization systems with the ceramic-composite membranes of the present work

Adsorbent (configuration)	Sulfur compound/model fuel	Initial S conc.	Operation mode; pressure	Capacity^a^	Rejection/regeneration	Reference
UiO-66@silica, S-3 (PVA-bound silica ceramic-composite membrane)	DBT in *n*-decane	50–200 mg S per L (100 baseline)	Recirculating cross-flow; 10–30 bar	25 mg S per g at 120 min (≈100 per g UiO-66)	62.5%; 88% over 5 cycles (methanol)	This work
UiO-66@alumina, A-3 (PVA-bound γ-alumina ceramic-composite membrane)	DBT in *n*-decane	50–200 mg S per L	Recirculating cross-flow; 10–30 bar	22.8 mg S per g at 120 min (≈96 per g UiO-66)	60%; 85% over 5 cycles (methanol)	This work
UiO-66 (powder)	DBT in fuel oil	n.r.	Batch (ambient)	22.0 mg S per g	—; n.r.	37
UiO-66-NH_2_ (powder)	Thiophene/benzothiophene in model fuel	n.r.	Batch	29.5 (TP); 35.7 (BT) mg S per g	—; Regenerable (ethanol)	23
Ionic-liquid-modified UiO-66 (Mod-UiO-66@IL1, powder)	DBT in model oil	2000 mg L^−1^	Batch	43.52 mg S per g	—; ∼Retained over 5 cycles (methanol)	38
UiO-66-NH_2_ tubular membrane	Thiophene/model gasoline	n.r.	Pervaporation (not adsorption)	n/a (Flux/enrichment)	—; n.r.	39

a Capacities are quoted per source: this work and several studies report mg S per g, others mg DBT per g; these differ by DBT's sulfur mass fraction (≈0.174), so cross-study comparison is approximate. Pressure/flux/rejection apply only to flow/membrane configurations (this work); powder studies are batch. n.r. = not reported; n/a = not applicable.

## 4. Conclusions

A silica- and alumina-based UiO-66 loading series (0, 1, 3, and 5 wt%) was prepared as flat-sheet PVA-bound ceramic-composite membranes. Simultaneous evaluation of adsorption capacity and sulfur rejection identified 3 wt% as the best-performing loading within the tested range, and the blanks (S-0 and A-0) and selected composites (S-3 and A-3) were carried forward for detailed characterization and operating-parameter studies. TGA, N_2_ physisorption, FTIR, XRD, and SEM confirmed UiO-66 incorporation in the selected composites without loss of the ceramic-host structure, with a moderate BET surface-area increase and added microporosity while type IV mesoporosity was retained.

UiO-66 incorporation further improved DBT/*n*-decane desulfurization at 25 °C on top of the already substantial ceramic-host capacity (Section 3.6.1), with higher rejection and a moderate flux penalty (Table 3). This benefit increased with pressure/concentration and decreased at higher flow rate, consistent with adsorption limited jointly by site availability and residence time (Sections 3.6.2–3.6.4). Over five cycles, the composites retained substantially more capacity than the blanks (Section 3.7 and Table 3).

S-3 was the most effective formulation overall, combining the highest surface area, capacity, rejection and cycling retention (Table 3), tentatively linked to more favorable UiO-66 dispersion on the silanol-rich silica surface (Section 3.5).

Embedding a benchmark Zr-MOF in a mesoporous ceramic-composite membrane offers a practical route toward a robust, continuous-flow adsorptive-desulfurization platform for a DBT/*n*-decane model fuel. Relevance to real middle-distillate streams remains to be confirmed; priority future work includes loading-specific morphological and textural characterization across an expanded loading range, breakthrough-curve and pre-equilibrated flux measurements, quantitative mechanical characterization, and multicomponent real-fuel evaluation.

## Author contributions

El-Sayed M. El-Sayed: conceptualization, methodology, experimental work, data curation, formal analysis, investigation, visualization, and writing—original draft. Hussien A. El Sayed: conceptualization, supervision, validation, and writing—review and editing. Ahmed M. A. El Naggar: supervision, methodology, validation, and writing—review and editing. M. A. Sayed: supervision, resources, validation, and writing—review and editing. Mahmoud F. Mubarak: conceptualization, supervision, methodology, validation, and writing—review and editing. Ahmed Shahat: resources, formal analysis, validation, and writing—review and editing. Reda M. Abd El-Aal: resources, validation, supervision, and writing—review and editing. All authors have read and approved the final version of the manuscript.

## Conflicts of interest

On behalf of all authors, the corresponding author states that there is no conflict of interest.

## Data Availability

The datasets generated and/or analyzed during the current study are available from the corresponding author on reasonable request.

## References

[cit1] Stanislaus A., Marafi A., Rana M. S. (2010). Recent advances in the science and technology of ultra low sulfur diesel (ULSD) production. Catal. Today.

[cit2] Babich I. V., Moulijn J. A. (2003). Science and technology of novel processes for deep desulfurization of oil refinery streams: a review☆. Fuel.

[cit3] Song C. (2003). An overview of new approaches to deep desulfurization for ultra-clean gasoline, diesel fuel and jet fuel. Catal. Today.

[cit4] Song C., Ma X. (2003). New design approaches to ultra-clean diesel fuels by deep desulfurization and deep dearomatization. Appl. Catal., B.

[cit5] Ahmed I., Jhung S. H. (2016). Adsorptive desulfurization and denitrogenation using metal-organic frameworks. J. Hazard. Mater..

[cit6] Fu R.-X. (2026). *et al.*, Metal-organic frameworks in adsorption desulfurization: Recent progress, new trends and future perspectives. Coord. Chem. Rev..

[cit7] Saeed M. (2023). *et al.*, MOFs for desulfurization of fuel oil: Recent advances and future insights. J. Chin. Chem. Soc..

[cit8] Shayanmehr M. (2025). *et al.*, A data driven machine learning approach for predicting and optimizing sulfur compound adsorption on metal organic frameworks. Sci. Rep..

[cit9] M R., El-Sayed E. M. (2025). Synthesis and characterization of Al-based metal–organic framework with superior performance for adsorptive desulfurization of model fuel. Sci. Rep..

[cit10] Wang C. (2025). *et al.*, The Facile Construction of Defect-Engineered and Surface-Modified UiO-66 MOFs for Promising Oxidative Desulfurization Performance. Nanomaterials.

[cit11] Tariq A. (2026). *et al.*, From metal nodes to sulfur capture: design principles and challenges in MOF-based adsorptive desulfurization. Dalton Trans..

[cit12] Vakil F., Shahid M. (2025). Unlocking the Potential of Metal–Organic Frameworks for Adsorptive Desulfurization of Fuels: Insights into Structure, Mechanism, and Performance. Energy Fuels.

[cit13] Hong-Cai Zhou J. R. L., Yaghi O. M. (2012). Introduction to Metal–Organic Frameworks. Chem. Rev..

[cit14] Furukawa H. (2013). *et al.*, The Chemistry and Applications of Metal-Organic Frameworks. Science.

[cit15] Cavka J. H. (2008). *et al.*, A New Zirconium Inorganic Building Brick Forming Metal Organic Frameworks with Exceptional Stability. J. Am. Chem. Soc..

[cit16] Valenzano L. (2011). *et al.*, Disclosing the Complex Structure of UiO-66 Metal Organic Framework: A Synergic Combination of Experiment and Theory. Chem. Mater..

[cit17] Kampouraki Z.-C. (2019). *et al.*, Metal Organic Frameworks as Desulfurization Adsorbents of DBT and 4,6-DMDBT from Fuels. Molecules.

[cit18] Khan N. A., Jhung S. H. (2012). Low-temperature loading of Cu+ species over porous metal-organic frameworks (MOFs) and adsorptive desulfurization with Cu+-loaded MOFs. J. Hazard. Mater..

[cit19] Yeskendir B. (2021). *et al.*, From metal–organic framework powders to shaped solids: recent developments and challenges. Mater. Adv..

[cit20] Liu X. (2015). *et al.*, Highly Water-Stable Zirconium Metal–Organic Framework UiO-66 Membranes Supported on Alumina Hollow Fibers for Desalination. J. Am. Chem. Soc..

[cit21] Wang X. (2023). *et al.*, A Mini Review of Ceramic-Based MOF Membranes for Water Treatment. Membranes.

[cit22] Zhang P. (2021). *et al.*, Hierarchical-pore UiO-66 modified with Ag+ for π-complexation adsorption desulfurization. J. Hazard. Mater..

[cit23] Zhang X.-F. (2018). *et al.*, Adsorptive desulfurization from the model fuels by functionalized UiO-66(Zr). Fuel.

[cit24] Park H. B. (2017). *et al.*, Maximizing the right stuff: The trade-off between membrane permeability and selectivity. Science.

[cit25] Schaate A. (2011). *et al.*, Modulated Synthesis of Zr-Based Metal–Organic Frameworks: From Nano to Single Crystals. Chem.–Eur. J..

[cit26] Shearer G. C. (2016). *et al.*, Defect Engineering: Tuning the Porosity and Composition of the Metal–Organic Framework UiO-66 via Modulated Synthesis. Chem. Mater..

[cit27] DeCoste J. B. (2013). *et al.*, Stability and degradation mechanisms of metal–organic frameworks containing the Zr6O4(OH)4 secondary building unit. J. Mater. Chem. A.

[cit28] Thommes M. (2015). *et al.*, Physisorption of gases, with special reference to the evaluation of surface area and pore size distribution (IUPAC Technical Report). Pure Appl. Chem..

[cit29] Li Y.-X. (2015). *et al.*, What Matters to the Adsorptive Desulfurization Performance of Metal-Organic Frameworks?. J. Phys. Chem. C.

[cit30] Katz M. J. (2013). *et al.*, A facile synthesis of UiO-66, UiO-67 and their derivatives. Chem. Commun..

[cit31] Kandiah M. (2010). *et al.*, Synthesis and Stability of Tagged UiO-66 Zr-MOFs. Chem. Mater..

[cit32] Hu Z. (2015). *et al.*, A Modulated Hydrothermal (MHT) Approach for the Facile Synthesis of UiO-66-Type MOFs. Inorg. Chem..

[cit33] Bai Y. (2016). *et al.*, Zr-based metal–organic frameworks: design, synthesis, structure, and applications. Chem. Soc. Rev..

[cit34] Trickett C. A. (2015). *et al.*, Definitive Molecular Level Characterization of Defects in UiO-66 Crystals. Angew. Chem., Int. Ed..

[cit35] Ren M. (2022). *et al.*, Dynamic simulation of adsorption desulfurization from diesel fuel over activated carbon in the fixed bed. Chem. Eng. Res. Des..

[cit36] Wang Y. (2012). *et al.*, Optimization of adsorptive desulfurization process of jet fuels for application in fuel cell systems. Fuel Process. Technol..

[cit37] Chen H. (2023). *et al.*, Dibenzothiophene Removal from Fuel Oil by Metal-Organic Frameworks: Performance and Kinetics. Int. J. Environ. Res. Public Health.

[cit38] Shan Q. (2022). *et al.*, Preparation of ionic liquid-type UiO-66 and its adsorption desulfurization performance. Fuel.

[cit39] Wu F. (2018). *et al.*, High-performance UiO-66-NH2 tubular membranes by zirconia-induced synthesis for desulfurization of model gasoline via pervaporation. J. Membr. Sci..

